# Adherence to ACSM exercise guidelines and its influence on Fibromyalgia treatment outcomes: a meta-analysis of randomized controlled trials

**DOI:** 10.3389/fphys.2024.1413038

**Published:** 2024-07-19

**Authors:** Tianran Han, Rui Xi, Jialin Wang, Huiqian Yan, Linhua Li

**Affiliations:** Sports Rehabilitation Research Center, China Institute of Sport Science, Beijing, China

**Keywords:** Fibromyalgia, exercise dosage, ACSM, meta-analysis, treatment outcomes

## Abstract

**Background:**

The Fibromyalgia Syndrome (FMS) is a multifaceted chronic pain disorder that exerts a substantial impact on the overall state of health and quality of life of patients.

**Purpose:**

Investigate the effects of exercise therapy and adherence to the American College of Sports Medicine (ACSM) guidelines on treatment outcomes in FMS patients.

**Methods:**

The literature search, which concluded in October 2023, encompassed studies investigating the impact of exercise interventions on patients diagnosed with FMS and providing adequate data for calculating standardized mean difference (SMD). The primary outcome measures encompassed the Fibromyalgia Impact Questionnaire (FIQ) and Health Assessment Questionnaire (HAQ), while secondary outcome measures comprised pain levels, sleep quality, fatigue, and mental health.

**Results:**

Among 4,008 records, 19 studies (patients = 857) were eligible for qualitative synthesis. The meta-analysis revealed that the SMD for overall state of health impact was −0.94 (95%CI −1.26, −0.63), and the pooled SMD for the subgroup with high adherence to ACSM guidelines was −1.17 (95%CI −1.65, −0.69). The SMD for the subgroup with low or uncertain adherence was −0.73 (95%CI −1.12, −0.34). The overall effects included a −1.21 (95%CI −1.62, −0.79) SMD for pain relief, with high adherence achieving a −1.32 (95%CI −2.00, −0.64) SMD and low adherence a −1.06 (95%CI −1.55, −0.57) SMD. Mental health improvements showed a −0.95 (95%CI −1.32, −0.57) overall SMD, with high and low adherence subgroups at −0.96 (95%CI −1.62, −0.30) and −0.94 (95%CI −1.29, −0.60), respectively. Sleep quality impact was −1.59 (95%CI −2.31, −0.87) overall, with high adherence at −1.71 (95%CI −2.58, −0.83) and low adherence at −1.11 (95%CI −1.88, −0.33). Fatigue impact had a −1.55 (95%CI −2.26, −0.85) overall SMD, with −1.77 (95%CI −3.18, −0.36) for high adherence and −1.35 (95%CI −2.03, −0.66) for low adherence.

**Conclusion:**

Exercise therapy can improve the overall state of health, pain, sleep, and fatigue of FMS patients, particularly when adhering to ACSM guidelines. However, adherence levels do not affect mental health gains, indicating a need for future research on psychological impact.

**Systematic Review Registration:**

https://inplasy.com/inplasy-2024-3-0106/, identifier INPLASY202430106.

## 1 Introduction

The Fibromyalgia Syndrome (FMS) is a chronic condition characterized by widespread, diffuse pain throughout the body, often accompanied by sleep disturbances, mood fluctuations, and cognitive changes (Bannwarth, et al., 2009; Branco, et al., 2010; Chinn, et al., 2016; Garrido-Ardila, et al., 2021). The FMS arises from a multitude of physiological mechanisms, such as central sensitization, peripheral mechanisms, and aberrant neural pain processing (Rodríguez-Almagro, et al., 2023). Central sensitization refers to an augmented responsiveness of the central nervous system to pain signals, resulting in heightened pain perception among patients (Staud, et al., 2007; Goubert, et al., 2017; Oliva, et al., 2022). Peripheral pain mechanisms involve aberrant signaling within muscles and soft tissues (Giesecke, et al., 2004; González-Roldán, et al., 2016). Furthermore, FMS patients frequently exhibit neurological abnormalities characterized by increased sympathetic activity and metabolic dysregulation in the hippocampus (Wood, et al., 2009). Initial studies (Wolfe, et al., 1995; Zhu, 2021) indicated a general population prevalence ranging from approximately 2%–4%, recent research highlights significant geographical variations and the influence of regional healthcare differences. For instance, Sarzi-Puttini et al. report higher prevalence rates in Western countries (Sarzi-Puttini, et al., 2020), contrasting sharply with much lower rates in China (with an outpatient diagnosis rate of 0.25%) (Zeng, et al., 2010) as noted by Zeng et al., cultural and systemic factors may affect the identification of the disease. Furthermore, factors such as increased societal stressors, accelerated pace of modern life, significant reduction in physical activity levels and unhealthy dietary habits have led to a significant rise in FMS incidence rates (Giorgi, et al., 2023; Lee, et al., 2024). Particularly noteworthy is a recent epidemiological study (Althobaiti, et al., 2022) conducted in Saudi Arabia which reported an alarming prevalence rate of 7.6%. This underscores both the high occurrence of this disease in specific regions and its substantial impact on patients’ quality of life and socioeconomic status while emphasizing the urgent need for exploring more effective treatment approaches.

In the realm of treatment strategies, drug therapy offers limited efficacy due to the diverse pathogenesis involved in FMS as well as concerns about potential side effects (Zhang, et al., 2022). However, exercise therapy has garnered significant attention due to its practicality, cost-effectiveness, and potential for long-term benefits (Macfarlane, et al., 2017; Maffei, 2020). Numerous studies have shown that exercise can relieve FMS symptoms through various mechanisms. Firstly, it enhances muscle strength and reduces muscle fatigue to alleviate pain (Estévez-López, et al., 2021). Secondly, exercise stimulates the production of endogenous opioids and β-endorphins which activate descending pain inhibition mechanisms, thus mitigating pain perception (Tan, et al., 2022). Thirdly, exercise promotes nerve growth, metabolite regulation, and improves central nervous system function, reducing fatigue levels while enhancing cognitive function (Valim, et al., 2013). Recent studies (Busch, et al., 2011) have consistently demonstrated that exercise therapy holds promise in alleviating symptoms such as pain, sleep disturbances, and depression among patients diagnosed with FMS, while also offering notable economic and safety advantages. For instance, a study (Larsson, et al., 2015) demonstrated significant enhancements in Fibromyalgia Impact Questionnaire (FIQ) scores and pain levels following a 15-week intervention incorporating a resistance exercise program. Similarly, the research team led by Ana Assumpção has validated the beneficial therapeutic effects of flexibility and resistance exercises on individuals with FMS (Assumpção, et al., 2018).

The recent systematic review and meta-analysis (Rodríguez-Almagro, et al., 2023) conducted to assess the effects of Physical exercise-based therapy (PEBT) on patients with FMS revealed statistically significant outcomes in terms of pain relief standardized mean differences (SMD) −0.62 (95%CI −0.78, −0.46) and improvement in mental health [SMD = 0.48 (95%CI 0.29, 0.67)]. Furthermore, it was concluded that an optimal PEBT dosage would consist of 21–40 sessions, administered three times weekly for a duration ranging from 31 to 60 mins. PEBT includes a large variety of aerobic, resistance, strength, balance, and proprioceptive exercises. Consequently, the study encompassed a diverse range of exercise patterns, exhibiting significant heterogeneity within groups. Moreover, there was considerable variation in the number of control groups incorporated in the studies due to limited availability for each type of control therapy. This potential scarcity may impact the overall quality of evidence derived from this study. In contrast, the American College of Sports Medicine (ACSM) provides comprehensive guidelines based on an extensive body of scientific research that is more systematic and standardized (Savin, et al., 2023; Xijuan et al., 2023; Zhian, et al., 2023). This guideline recommends standard forms of exercise that include aerobic, flexibility, and resistance exercises, with well-defined intensity levels for disease management (Richards, et al., 2003; Álvarez-Gallardo, et al., 2019; Thompson, et al., 2020; Yapeng, et al., 2021). However, some studies, such as the systematic review conducted by Busch and Alvarez-Gallardo, have demonstrated that exercise therapy recommended by the ACSM effectively alleviates symptoms in patients with FMS(Álvarez-Gallardo, et al., 2019; Busch, et al., 2007). Specifically, Alvarez-Gallardo’s study highlighted that the exercise intervention program recommended by the ninth edition ACSM exhibited reduced efficacy in treating FMS. This observation was attributed to incomplete descriptions of exercise intervention protocols and adherence in randomized controlled trials included within the review. Moreover, the latest ACSM guidelines exhibit certain modifications in comparison to the ninth edition, including a reduction in the recommended frequency of aerobic exercise from 2–4 times per week to 1–3 times per week, as well as a change in the suggested frequency of flexibility exercises from 1–3 times per week progressing to 5 times per week, to now being advised at 2–3 times per week. Consequently, despite the broad rationale (Thompson, et al., 2013; Thompson, et al., 2020), empirical research on the actual effectiveness of the most recent ACSM guidelines for FMS management remains relatively limited, particularly regarding the impact of exercise intervention adherence on treatment outcomes.

Hence, the objective of this study is to conduct a comprehensive assessment of the impact that adherence to the latest exercise guidelines recommended by ACSM has on treatment outcomes for patients with FMS. This will be accomplished through a systematic review and meta-analysis of existing literature. The ultimate aim is to establish a basis for developing more precise and personalized exercise prescriptions for clinical management.

## 2 Materials and methods

### 2.1 Search strategy

The Preferred Reporting Items for Systematic Reviews and Meta-Analyses (PRISMA) standards were followed for conducting this meta-analysis. The protocol for this review has been registered with the International Platform of Registered Systematic Review and Meta-analysis Protocols (INPLASY) under the registration number INPLASY202430106. The registration details can be accessed at https://inplasy.com/inplasy-2024-3-0106/. Literature searches were independently conducted by two researchers from the establishment of the database up until October 2023. Computer searches were performed in various databases including PubMed, Embase, Cochrane Library and Web of Science. Our search strategy adhered to the PICOS principles with a focus on study subjects’ interventions and research methodologies. The detailed search strategy is presented in Table 1.

**TABLE 1:** PubMed literature search strategy.

	Search strategy
#1	“Fibromyalgia" [MeSH Terms]
#2	(((((((((((((((Fibromyalgias [Title/Abstract]) OR (Fibromyalgia Fibromyositis Syndrome [Title/Abstract])) OR (Rheumatism, Muscular [Title/Abstract])) OR (Muscular Rheumatism [Title/Abstract])) OR (Fibrositis [Title/Abstract])) OR (Myofascial Pain Syndrome, Diffuse [Title/Abstract])) OR (Diffuse Myofascial Pain Syndrome [Title/Abstract])) OR (Fibromyositis Fibromyalgia Syndrome [Title/Abstract])) OR (Fibromyalgia, Secondary [Title/Abstract])) OR (Fibromyalgias, Secondary [Title/Abstract])) OR (Secondary Fibromyalgia [Title/Abstract])) OR (Secondary Fibromyalgias [Title/Abstract])) OR (Fibromyalgia, Primary [Title/Abstract])) OR (Fibromyalgias, Primary [Title/Abstract])) OR (Primary Fibromyalgia [Title/Abstract])) OR (Primary Fibromyalgias [Title/Abstract])
#3	#1 OR #2
#4	“Exercise" [MeSH Terms]
#5	((((((((((((((((((((((((Exercises [Title/Abstract]) OR (Physical Activity [Title/Abstract])) OR (Physical Activity [Title/Abstract])) OR (Activity, Physical [Title/Abstract])) OR (Physical Activities [Title/Abstract])) OR (Exercise, Physical [Title/Abstract])) OR (Exercises, Physical [Title/Abstract])) OR (Physical Exercise [Title/Abstract])) OR (Physical Exercises [Title/Abstract])) OR (Acute Exercise [Title/Abstract])) OR (Acute Exercises [Title/Abstract])) OR (Exercise, Acute [Title/Abstract])) OR (Exercises, Acute [Title/Abstract])) OR (Exercise, Isometric [Title/Abstract])) OR (Exercises, Isometric [Title/Abstract])) OR (Isometric Exercises [Title/Abstract])) OR (Isometric Exercise [Title/Abstract])) OR (Isometric Exercise [Title/Abstract])) OR (Aerobic Exercise [Title/Abstract])) OR (Aerobic Exercises [Title/Abstract])) OR (Exercises, Aerobic [Title/Abstract])) OR (Exercise Training [Title/Abstract])) OR (Exercise Trainings [Title/Abstract])) OR (Training, Exercise [Title/Abstract])) OR (Trainings, Exercise [Title/Abstract])
#6	#4 OR #5
#7	#3 AND #6

### 2.2 Eligibility criteria

The inclusion and exclusion criteria are shown in Table 2.

**TABLE 2:** Inclusion and exclusion criteria.

	Inclusion criteria	Excluded criteria
Clinical study	• Randomized controlled trials • Study subjects were FMS patients • The experimental group intervention could encompass any type of exercise, including resistance training, aerobic exercise, flexibility exercise, etc • Control interventions could involve no treatment or traditional treatment, such as conventional medication or care • Outcome measures in the study encompassed overall state of health, pain, sleep quality, fatigue, and mental health, including FIQ, VAS (Visual Analog Scale), SF-36 (36-Item Short Form Health Survey), BDI (Beck Depression Inventory), and others	• Studies reported as conference abstracts, review articles, or editorials • Participants with other chronic diseases, especially those related to chronic pain • Studies that administered other treatments during the exercise intervention, such as drugs, relaxation therapy, or specific sports (e.g., tai chi, Pilates, aquatic exercises) • Duplicate publications reporting the same experimental data from a single study • Unclear details of the intervention

### 2.3 Study selection

The titles and abstracts of the retrieved literature were independently assessed by two reviewers to determine inclusion criteria. If either reviewer considered a study potentially meeting the criteria, the full text of the article was obtained. Subsequently, both reviewers independently evaluated whether the full text met inclusion criteria. In cases of disagreement, a third reviewer facilitated final decision-making through discussion until consensus was reached. There were no restrictions on participant age, gender, body mass index, publication date or language.

### 2.4 Quality assessment

The quality of the included studies was assessed by two pairs of authors using the Cochrane Collaboration’s recommended quality evaluation standard for randomized controlled trials (Review Manager 5.4). The assessment encompassed random sequence generation, allocation concealment, blinding of participants and personal, blinding of outcome assessment, incomplete outcome data, selective reporting, other bias. Reviewers scored individual studies based on the Cochrane Handbook’s guidelines, categorizing the risk of bias in each domain as “low risk,” “some concerns,” or “high risk.” If all domains were deemed low risk, the overall risk of bias was considered low; if some domains raised “some concerns” and none were rated as high risk, the overall risk of bias was labelled as “some concerns”; and if any single domain was rated as “high risk,” the overall risk of bias was deemed “high risk."

### 2.5 Data extraction and analysis

The data extraction process was independently conducted by two authors. In this study, primary outcomes included FIQ and Health Assessment Questionnaire (HAQ), which reflect the overall state of health, while secondary outcomes comprised pain, sleep quality, fatigue, and mental health. An Excel spreadsheet was prepared in advance to extract relevant data, covering publication characteristics (author names, publication year, and country), methodological characteristics (number of study groups, group designs, interventions, and sample sizes), participant characteristics (age, sex ratio), exercise details (intervention frequency, intensity, duration, repetition numbers, and set numbers), as well as risk assessment and outcome features. The Webplotdigitizer software was used to extract data when outcome data were presented graphically without clear textual descriptions. The exercise dosage and adherence were subsequently evaluated according to the ACSM recommendations for patients with FMS. Each aspect of the exercise intervention in each study was independently scored by two authors according to the criteria defined by the ACSM (the 11th Edition) (Liguori, et al., 2021) recommended dosage, in order to assess exercise intervention adherence (see Table 3). The scoring ranged from 0 to 2 points for each exercise indicator, with 2 points indicating high adherence or fully meeting the criteria, 1 point indicating partial adherence or uncertainty, and 0 points indicating non-adherence or not meeting the criteria. In cases of disagreement, a discussion was held with a third author to reach a consensus. Using this scoring system, the proportion of exercise dosage adherence in each study according to the ACSM recommended dosage was calculated. A proportion of ≥75% was classified as high adherence with ACSM recommendations, while a proportion of <75% was classified as low or uncertain adherence.

**TABLE 3:** ACSM recommended dosage adherence assessment criteria for exercise intervention in FMS.

	Aerobic	Resistance	Flexibility
Frequency	1-3 days/wk	2-3 days/wk	2-3 days/wk
Intensity	moderate intensity, 30%–60% VO_2_R or HRR	Begin with 40%–80%1-RM, Gradually increase to 60%–80%1-RM for strength. Use≤50%1-RM For endurance	Stretch until you feel your muscles being pulled tight or a slight discomfort
Time	Begin with 10 min/d and progress to a total of 30–60 min/d	Strength: Begin with 4–5 repetitions, Gradually increase to 8–12 repetitions; 2–4 sets Endurance:15–20 repetitions, from 1 to 2 sets	Hold each stretch for 10–30s

Note: 1-RM, one repetition maximum; HRR, heart rate reserve; VO_2_R, oxygen uptake reserve.

We conducted this meta-analysis using Stata 15.0 to compare the outcomes of the selected studies. These studies were divided into two groups based on their adherence to the ACSM guidelines, categorized as high, low, or indeterminate adherence. To assess the heterogeneity within each subgroup, we employed the Higgins I^2^ statistic following the guidelines outlined in the Cochrane Handbook. A fixed-effects model was applied when the I^2^ value was 50% or less, while a random-effects model was utilized for I^2^ values exceeding 50%. The effect size was expressed as the SMD along with its corresponding 95% Confidence Interval (95% CI). We examined publication bias by generating a funnel plot to explore the relationship between each study’s effect size and its standard error. To assess funnel plot asymmetry, we conducted sensitivity analysis using Stata 15.0, progressively excluding studies to assess the robustness of our results.

## 3 Results

### 3.1 Study selection

**FIGURE 1:**
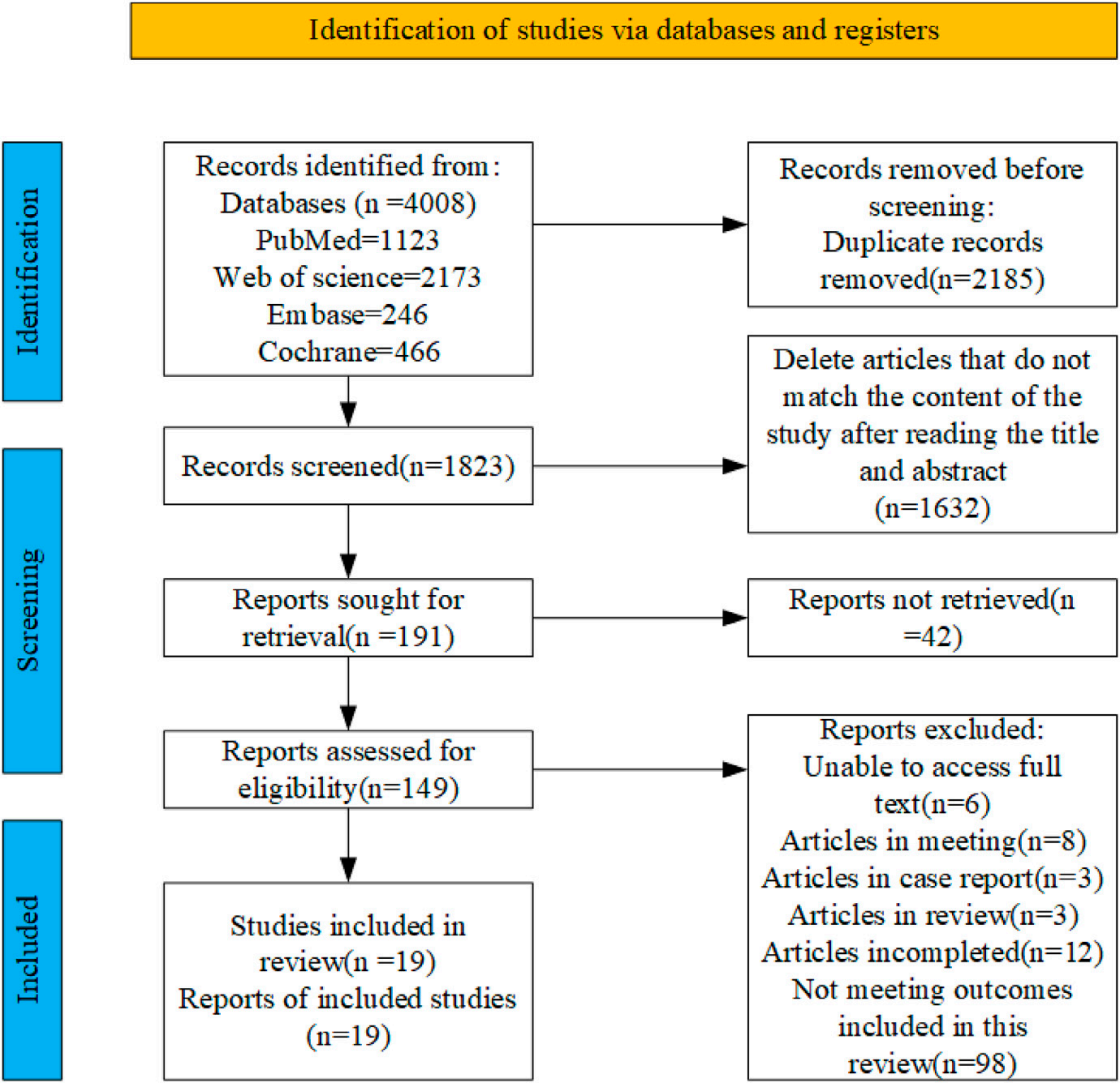
PRISMA study flow diagram.

A total of 4,008 articles were retrieved from four databases: PubMed (n = 1,123), Embase (n = 246), Web of Science (n = 2,173), and Cochrane (n = 466). After removing duplicates, 1823 records remained. Subsequently, following a thorough review of the titles and abstracts, 149 articles were identified as potential candidates for inclusion. Finally, after a comprehensive examination of the full texts, 19 relevant articles were included, the flowchart illustrating the literature selection process is presented in Figure 1.

### 3.2 Study characteristics

The 19 included articles encompassed 22 comparative research studies, with 3 articles reporting on two exercise intervention groups. In total, 857 participants were involved across the 22 studies, with 421 participants in the intervention group and 436 participants in the control group. Geographically, seven studies originated from Spain, three from Turkey and Brazil each, and two each from Italy and the USA, while Finland, the Netherlands, Iran, Sweden, and Northern Ireland each contributed one study. The duration of interventions across the 22 studies ranged from 5 weeks to 18 months. Regarding exercise dosage based on ACSM recommendations, 15 studies addressed aerobic exercise, 14 studies focused on resistance exercise, and 13 studies involved flexibility exercise. The characteristics of the included studies are outlined in Table 4.

**TABLE 4:** Basic characteristics of the included studies.

Author year	Country	Sample size(n) Total/male/female		Age (years) mean (SD)		BMI (kg/m^2^) mean (SD)		Intervention (Length of Intervention/Frequence/Duration)	Control	Outcome (overall state of health/Pain/Mental health/Sleep quality/Fatigue)
		IG	CG	IG	CG	IG	CG			
Sencan, et al. (2004)	Turkey	20/0/20	20/0/20	35.40 (9.62)	35.55 (7.86)	24.14 (3.73)	24.60 (2.64)	Aerobic exercises 6 weeks/3 times a week/40 mins	CON (TENS) Freq: 3 times a week Duration:6 weeks	VAS/BDI
Glasgow, et al. (2017)	USA	25/0/25	9/0/9	52 (13)	49 (8)	32.5 (4.4)	30.9 (3.9)	Resistance exercise Length of Intervention: 8 weeks Freq: twice a week	CON	FIQ/PCS
Larsson, et al. (2015)	Sweden	67/0/67	63/0/63	50.81 (9.05)	52.10 (9.78)	27.39 (5.29)	28.66 (5.32)	Resistance exercise 15 weeks/twice a week/60 mins (10 min warm up, 50 min standardized)	CON (Relaxation therapy)	FIQ/SF-36/PDI/PGIC /VAS/CPAQ/MCS/FABQ
Assumpção, et al. (2018)	Brazil	14/0/14	14/0/14	47.9 (5.3)	46.9 (6.5)	28.9 (4.2)	29.4 (4.8)	Flexibility exercise Length of Intervention: 12 weeks Freq: twice a week	CON (Usual medical treatment)	FIQ/SF-36/VAS
Assumpção, et al. (2018)	Brazil	16/0/16	14/0/14	45.7 (7.7)	46.9 (6.5)	28.1 (4.7)	29.4 (4.8)	Resistance exercise Length of Intervention: 12 weeks Freq: twice a week	CON (Usual medical treatment)	FIQ/SF-36/VAS
Atan, et al. (2020)	Turkey	19/0/19	17/0/17	46.57 (9.41)	52.70 (8.96)	NR	NR	T(H): HIIT 6weeks/5times a week/40 mins	CON (Usual medical treatment)	FIQ/SF-36/VAS
Atan, et al. (2020)	Turkey	19/0/19	17/0/17	47.36 (8.01)	52.70 (8.96)	NR	NR	T(M): MICT 6weeks/5times a week/40mins	CON (Usual medical treatment)	FIQ/SF-36/VAS
Estrada-Marcén, et al. (2023)	Spain	29/0/29	27/0/27	46.9 (9.7)	48.7 (7.8)	26.71 (3.90)	26.64 (4.15)	Aerobic exercises 16 weeks/3times a week/60 mins	CON(TAU)	FIQ//EQ-5D
Häkkinen, et al. (2001)	Finland	11/0/11	10/0/10	39 (6)	37 (5)	NR	NR	Resistance exercise Length of Intervention: 21 weeks Freq: twice a week	CON	HAQ/VAS/Depression index
Hernando-Garijo, et al. (2021)	Spain	17/0/17	17/0/17	51.81 (9.05)	55.06 (8.51)	27.25 (7.30)	25.93 (5.27)	Aerobic exercise 15 week/twice a week 50 mins	CON	FIQ/VAS/PCS/HADS
Sañudo, et al. (2010)	Spain	18/0/18	20/0/20	55.9 (1.6)	56.6 (1.9)	29.6 (1.1)	29.7 (1.1)	T(A): Aerobic exercise Freq: twice a week Duration: 45–60 mins	CON	FIQ/SF-36/BDI
Sañudo, et al. (2010)	Spain	17/0/17	20/0/20	55.9 (1.7)	56.6 (1.9)	27.6 (1.1)	29.7 (1.1)	T(C): Combined exercise Freq: twice a week Duration: 45–60 mins	CON	FIQ/SF-36/BDI
Izquierdo-Alventosa, et al. (2020)	Spain	16/0/16	16/0/16	53.06 (8.4)	55.13 (7.35)	NR	NR	Aerobic and Low-intensity physical exercise program 8 weeks/twice a week/60 mins	CON	FIQ/CPAQ-FM/PCS/HADS/BDI-II/PSS-10
Kingsley, et al. (2005)	USA	15/0/15	14/0/14	45 (9)	47 (4)	30.3 (6.4)	32.0 (7.8)	Resistance exercise Length of Intervention: 12 weeks Freq: twice a week	CON	FIQ
Norouzi, et al. (2020)	Iran	20/0/20	20/0/20	35.5 (2.42)	35.4 (2.80)	NR	NR	Aerobic exercise 12 weeks/3 times a week/60 mins	CON	BDI-II
Paolucci, et al. (2016)	Italy	18/0/18	18/0/18	50.4 (8.6)	51.3 (9.0)	24.7 (3.7)	23.8 (5.0)	Physical exercise 5 weeks/twice a week/60 mins	CON	FIQ
Paolucci, et al. (2015)	Italy	19/0/19	18/0/18	50.1 (8.9)	48.1 (10.4)	24.6 (3.8)	24.4 (5.1)	Physical exercise 5 weeks/twice a week/60 mins	CON	FIQ
Rodríguez-Mansilla, et al. (2021)	Spain	33/0/33	29/0/29	NR	NR	NR	NR	Active exercise 4 weeks/twice a week/45 mins	CON	FIQ/VAS
Sañudo, et al. (2011)	Spain	18/0/18	20/0/20	55.48 (7.14)	56.15 (8.48)	28.19 (5.1)	29.03 (5.4)	Combined aerobic and muscle strength training exercises 24 weeks/twice a week/45–60 mins	CON (Usual medical treatment)	FIQ/SF-36/BDI
Sañudo, et al. (2012)	Spain	13/0/13	12/0/12	NR	NR	NR	NR	Combined aerobic and muscle strength training exercises 3 years/twice a week/45–60 mins	CON	FIQ/SF-36/BDI
Schulze, et al. (2023)	Brazil	11/0/11	12/0/12	45.92 (7.33)	45.17 (11.77)	27.77 (4.9)	29.5 (5.7)	Flexibility exercise 8 weeks/1 time a week/40–45 mins	CON	FIQ/VAS
van Eijk-Hustings, et al. (2013)	Netherlands	19/0/19	48/0/48	43.9 (7.6)	42.9 (11.0)	NR	NR	Aerobic exercise 12 weeks/twice a week/60 mins	CON	FIQ

Note: IG: Intervention Group; CG: Control Group; CON: Control; VAS: Visual Analog Scale; BDI: Beck Depression Inventory; NR: Not reported; PCS: Pain Catastrophizing Scale; FIQ: Fibromyalgia Impact Questionnaire; CPAQ: Chronic Pain Acceptance Questionnaire; HIIT: High Intensity Interval Training; MICT: Moderate Intensity Continuous Training; PGIC: Patient global impression of change; TAU: Treatment-as-usual control arm; EQ-5D: EuroQol Five Dimensions Questionnaire; HADS: Hospital Anxiety and Depression Scale; SF-36: The medical outcomes study 36-item short from health survey; BDI-II: The Beck Depression Inventory-II; PSS-10: Perceived Stress Scale-10; IPQ-R: Illness Perception Questionnaire; TENS: Transcutaneous Electrical Nerve Stimulation;HAQ: Health Assessment Questionnaire; MCS: Mental component scale; FABQ: The Fear Avoidance Beliefs Questionnaire.

### 3.3 Risk of bias

**FIGURE 2:**
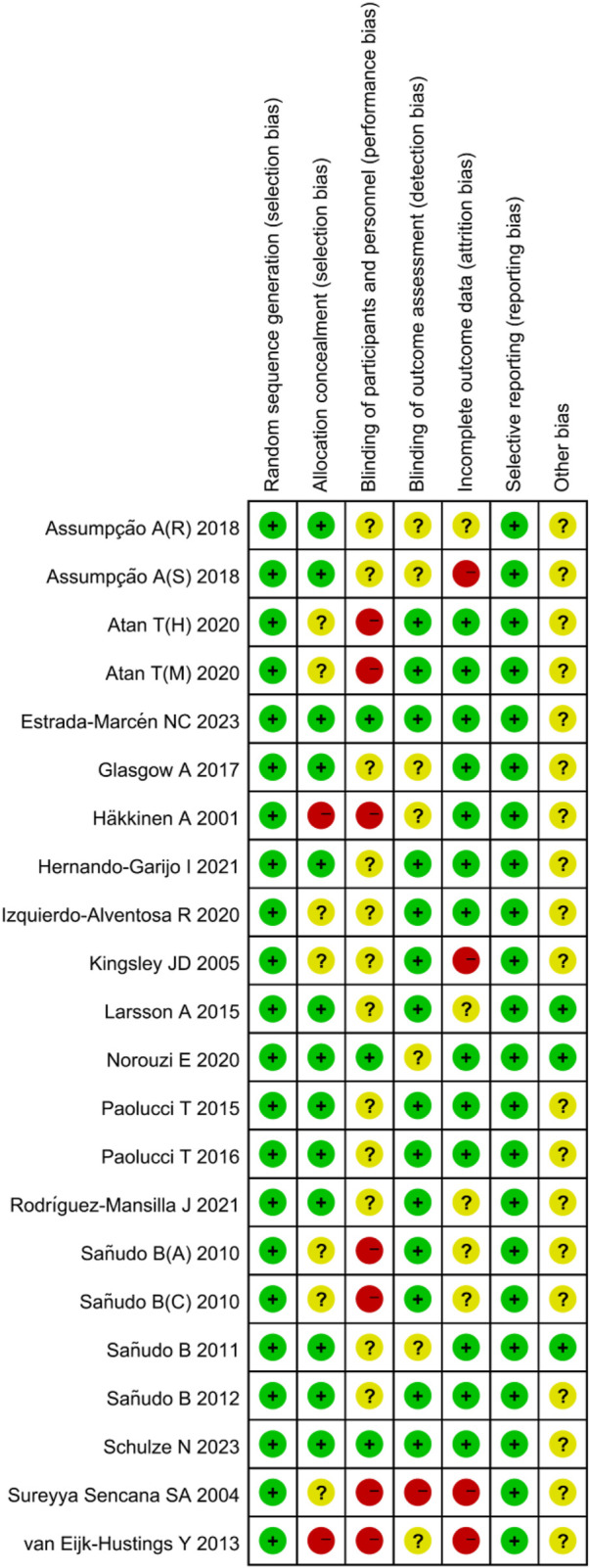
Risk of bias summary.

**FIGURE 3:**
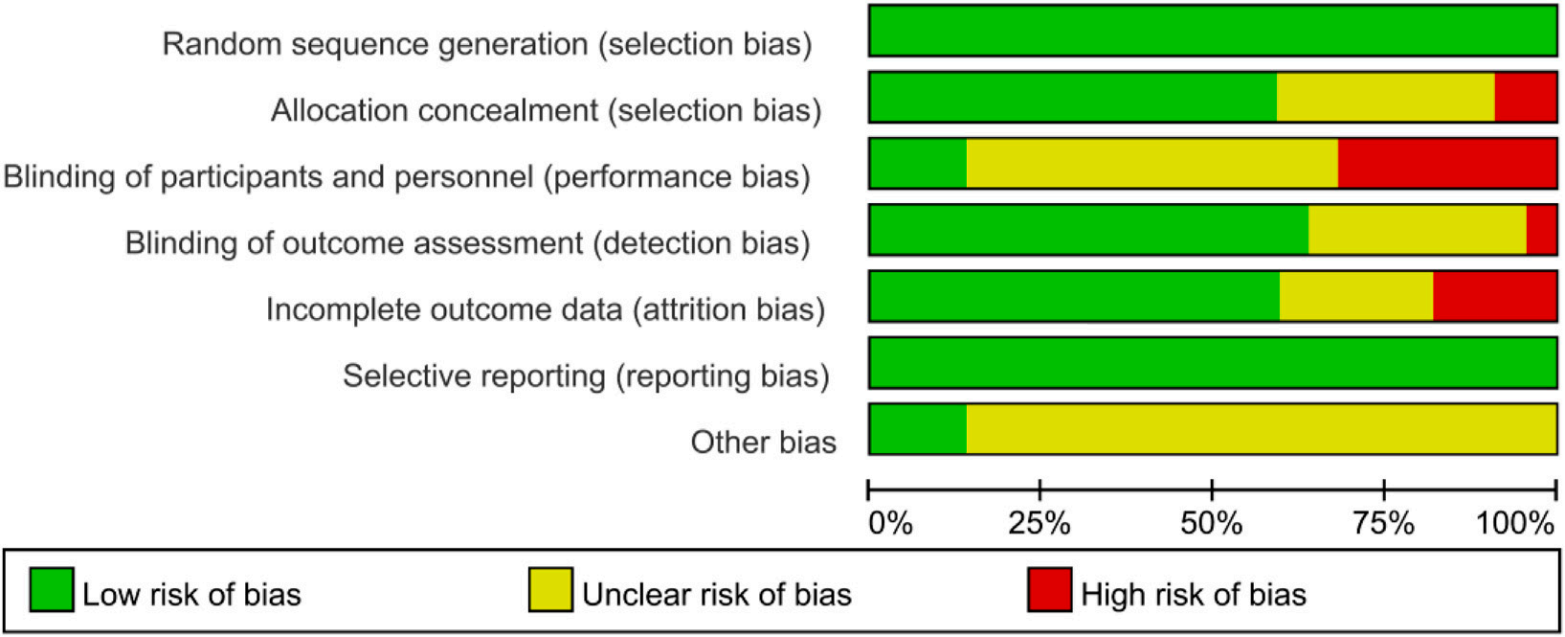
Risk of bias graph.

All studies indicated a low risk of bias in random sequence generation. Out of the 19 included studies, 12 were deemed to have a low risk of allocation concealment bias, while five did not report the allocation method, leading to an uncertain risk. Two studies were identified as having a high risk of allocation concealment bias. Blinding of both researchers and participants posed challenges due to the difficulty of implementing exercise interventions in a double-blind manner, resulting in a relatively high overall risk of bias in this category. Regarding outcome assessment blinding, 12 studies utilized blinded assessors, indicating a low risk, while six studies did not specify the outcome assessment method, raising some concerns, and one study lacked outcome blinding, hence classified as having a high risk. Among the 15 studies with incomplete outcome reporting, consistency in the number of subjects post-intervention compared to baseline was observed in most, leading to a low risk assessment. In 12 studies, the dropout rate was small (≤4 individuals), raising some concerns, while four studies experienced significant differences in subject numbers pre and post-intervention (≥7 individuals), resulting in a high risk assessment. The risk of selective reporting bias was low across the studies, and there were no high risks of other biases identified (Figures 2, 3).

### 3.4 Meta-analyses

Adherence with the ACSM recommendations was observed in 12 out of 22 results (3 studies were divided into two results), achieving a adherence rate of ≥75%. In the remaining 10 results, adherence with ACSM was less than 75%. The primary reasons for low adherence included discrepancies between the exercise intervention dosage and the ACSM recommendations, as well as insufficient information on exercise prescription for proper evaluation. When considering outcome measures, the proportions of adherence were analyzed as follows: For studies with the overall state of health as the outcome measure, 10 results demonstrated high adherence to ACSM guidelines, while 10 studies exhibited low or uncertain adherence. For studies measuring pain outcomes, eight showed high adherence to ACSM guidelines, while 6 had low or uncertain adherence. For studies focusing on mental health outcomes, 8 displayed high adherence to ACSM guidelines, while seven had low or uncertain adherence. Regarding sleep quality outcomes, four achieved high adherence to ACSM guidelines compared to only one study demonstrating low or uncertain adherence. As for fatigue outcomes, there were four studies that adhered well to ACSM guidelines and an equal number that had low or uncertain adherence. The detailed results are shown in Table 5.

**TABLE 5:** Exercise interventions evaluated according to the ACSM recommendations.

Author year	Aerobic exercise						Resistance exercise								Flexibility exercise						ACSM adherence	
	Frequency/Adherence		Intensity/Adherence		Duration/Adherence		Frequency/Adherence		Intensity/Adherence		Repetitions/Adherence		Sets/Adherence		Frequency/Adherence		Intensity/Adherence		Duration/Adherence		Points	Percent
Larsson, et al. (2015)							2 days/wk	J	40% of 1 RM, 3–4 weeks: 60% of 1 RM, 6–8 weeks: 80% of 1RM	J	Begin with15-20reps; 3–4week:10–12reps 6-8weks:5-8reps	L	1–2 sets	K	2 days/wk	J	NR	K	NR	K	9/14	64%
Assumpção, et al. (2018)							2 days/wk	J	0.5 kg was added each week if the patient identified the effort as slightly intense on the Borg scale	L	8reps	J	NR	K							5/8	63%
Assumpção, et al. (2018)															2 days/wk	J	Gradually to the point of moderate discomfort	J	30 s/time	J	6/6	100%
Sencan, et al. (2004)	3 days/wk	J	NR	K	40min	J															5/6	83%
(Atan, et al., 2020)	5 days/wk	L	80%–95% of peak HR 70% of peak HR	L	35min	J	5 days/wk	L	1–3 kg	L	8–10reps	J	1 set	L	5 days/wk	L	NR	K	20–30 s/time	J	7/20	35%
(Atan, et al., 2020)	5 days/wk	L	65%–70% of peak HR	K	55min	J	5 days/wk	L	1–3 kg	L	8–10reps	J	1 set	L	5 days/wk	L	NR	K	20–30 s/time	J	8/20	40%
Estrada-Marcén, et al. (2023)	3 days/wk	J	80% of the maximum heart rate	L	40min	J	3 days/wk	J	NR	K	20reps	J	1 set	L							9/14	64%
Glasgow, et al. (2017)							2 days/wk	J	50%–60% 1RM	J	8–12reps	J	3 sets	J							8/8	100%
Häkkinen, et al. (2001)							2 days/wk	J	1–3week:40%–60% 1RM 4–7week:60%–70% 1RM 8–14week:60%–80% 1RM 15–21week:70%–80% 1RM	J	15–20 reps 10–12 reps 8–12 reps 5–10 reps	K	NR	K							6/8	75%
Hernando-Garijo, et al. (2021)	2 days/wk	J	The repetition rate determines the intensity of the exercises	L	50min	J															4/6	67%
Izquierdo-Alventosa, et al. (2020)	2 days/wk	J	CR-10 Borg:1–2 CR-10 Borg:3–4	L	60min	J	2 days/wk	J	Endurance: 0.5–3 kg	J	15–20 reps	J	NR	K	2 days/wk	J	NR	K	NR	K	15/20	75%
Kingsley, et al. (2005)							2 days/wk	J	40%–80% 1RM	J	8–12 reps	J	1 set	L	2 days/wk	J	NR	K	NR	K	10/14	71%
Norouzi, et al. (2020)	3 days/wk	J	60%–75% estimated maximum heart rate	K	60min	J															5/6	83%
Paolucci, et al. (2016)	2 days/wk	J	40%–60% estimated maximum heart rate	J	20min	K															5/6	83%
Paolucci, et al. (2015)	2 days/wk	J	60% estimated maximum heart rate	K	20min	K	2 days/wk	J	NR	K	10reps	J	3 sets	J	2 days/wk	J	NR	K	30–60 s/time	K	15/20	75%
Rodríguez-Mansilla, et al. (2021)	2 days/wk	J	NR	K	45min	J									2 days/wk	J	NR	K	10 s/time	J	10/12	83%
Sañudo, et al. (2012)	2 days/wk	J	65%–70% HRmax	K	10–15min	K	2 days/wk	J	1–3 kg	L	8–10reps	L	1 set	L	2 days/wk	J	NR	K	30 s/time	J	11/20	55%
Sañudo, et al. (2011)	2 days/wk	J	65%–70% HRmax	K	10–15min	K	2 days/wk	J	1–3 kg	L	8–10reps	L	1 set	L	2 days/wk	J	NR	K	30 s/time	J	11/20	55%
Sañudo, et al. (2010)	2 days/wk	J	60%–65% HRmax 75%–80% HRmax	K	30–35min	J															5/6	83%
Sañudo, et al. (2010)	2 days/wk	J	60%–65% HRmax 75%–80% HRmax	K	30–35min	J	2 days/wk	J	1–3 kg	L	8–10reps	L	1 set	L	2 days/wk	J	NR	K	30 s/time	J	12/20	60%
Schulze, et al. (2023)															2 days/wk	J	The flexibility continued until the first sensation of tissue resistance	J	30 s/time	J	6/6	100%
van Eijk-Hustings, et al. (2013)	2 days/wk	J	55%–64% of the predicted maximum heart rate	J	30min	J	2 days/wk	J	NR	K	NR	K	NR	K	2 days/wk	J	NR	K	NR	K	15/20	75%

Note: J: high adherence or fully meeting the criteria; K: partial adherence or uncertainty; L: Non-adherence or not meeting the criteria; Reps: repetitions.

#### 3.4.1 Overall state of health

**FIGURE 4:**
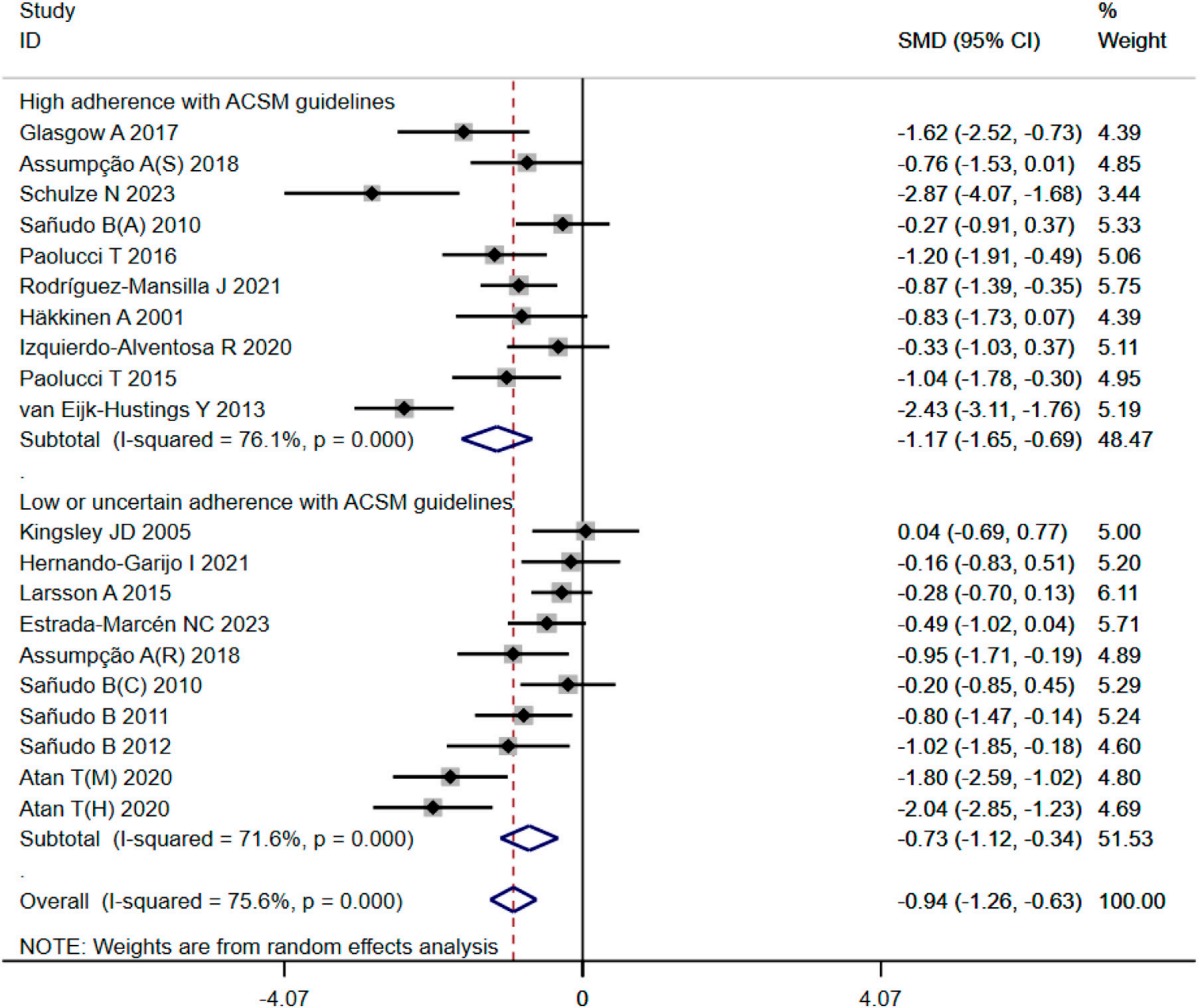
Forest plot of meta-analysis on the effect of exercise intervention and ACSM adherence on overall state of health in FMS patients.

In our analysis of 20 studies involving 777 participants, we investigated the impact of exercise interventions on the overall state of health and pain levels of patients with FMS. Upon conducting a heterogeneity test, we observed significant variability among the studies (I^2^ = 75%, *p* < 0.00001). Therefore, we employed a random effects model for statistical analysis. The analysis revealed a total combined SMD of −0.94 (95%CI −1.26, −0.63), indicating a beneficial effect of exercise intervention on the overall state of health of FMS patients. To further explore the differences in outcomes based on adherence with the ACSM recommendations, we performed subgroup analysis, categorizing the studies into two groups: high ACSM adherence and low or uncertain ACSM adherence. The combined SMD for the subgroup with high adherence was −1.17 (95%CI −1.65, −0.69), while for the subgroup with low or uncertain adherence, the combined SMD was −0.73 (95%CI −1.12, −0.34). Subgroup difference analysis demonstrated a distinction between exercise interventions with high ACSM adherence and those with low or uncertain ACSM adherence (Figure 4). From these results, we can conclude that exercise interventions with high ACSM adherence have better therapeutic effects for FMS patients compared to those with low or uncertain ACSM adherence. In terms of heterogeneity within the subgroups, we observed individual study heterogeneity of 76% in the high ACSM adherence subgroup and 71.6% in the low or uncertain ACSM adherence subgroup. Visual inspection of the funnel plot (Figure 9A) indicated approximate symmetry on both sides, suggesting the absence of publication bias. Sensitivity analysis (Figure 10A) demonstrated that no single study significantly influenced the overall results, confirming the robustness of our findings.

#### 3.4.2 Pain

**FIGURE 5:**
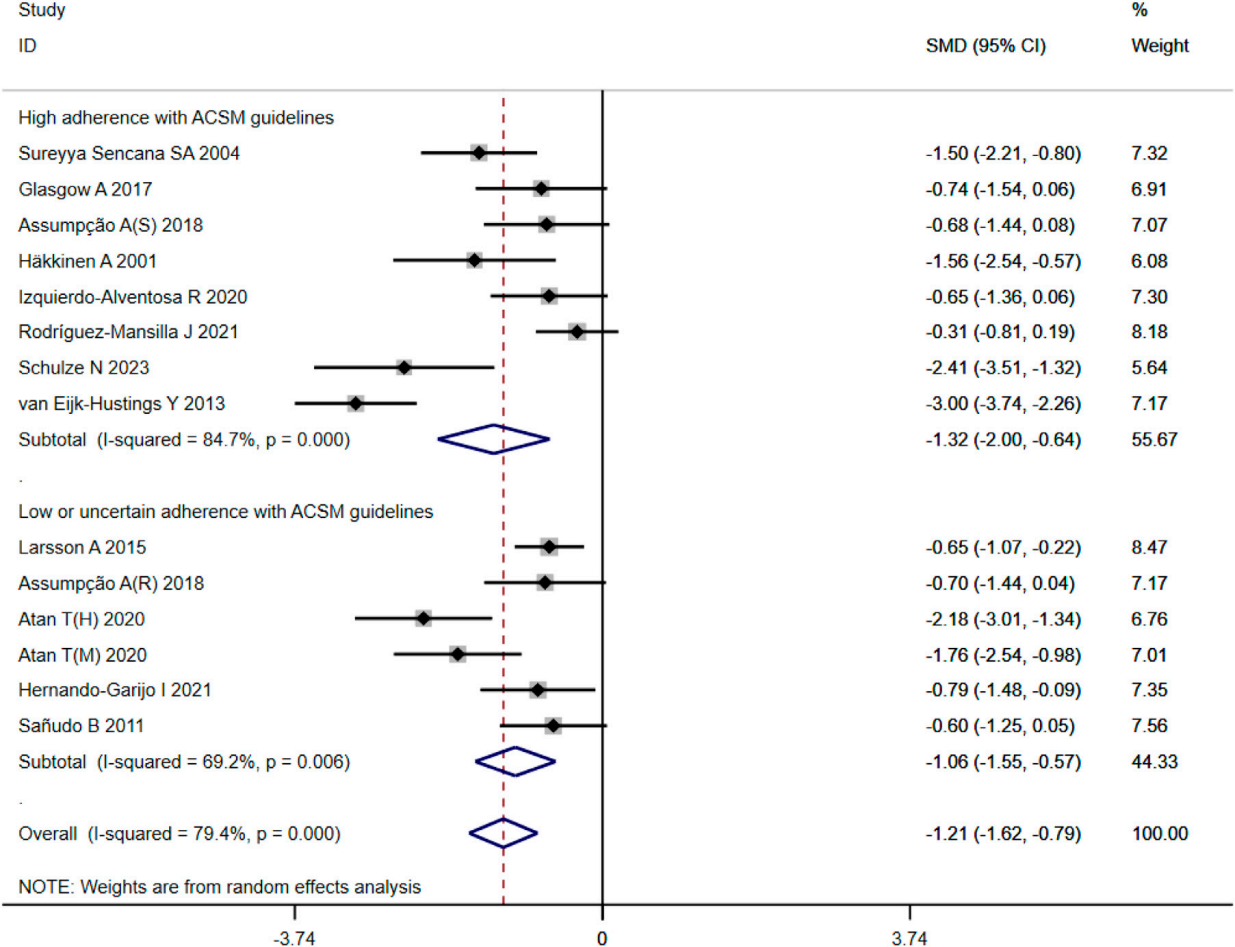
Forest plot of meta-analysis on the effect of exercise intervention and ACSM adherence on pain in FMS patients.

In the 14 studies with 564 participants that focused on pain as the outcome measure, we encountered a similar heterogeneity issue (I^2^ = 79%, *p* < 0.00001), prompting us to use a random effects model for statistical analysis. The analysis revealed a total combined SMD of −1.21 (95% CI −1.62, −0.79), indicating a beneficial effect of exercise intervention on pain relief in FMS patients. Subgroup analysis based on adherence with ACSM recommendations revealed a combined SMD of −1.32 (95% CI −2.00, −0.64) for the high ACSM adherence subgroup and −1.06 (95% CI −1.55, −0.57) for the low or uncertain ACSM adherence subgroup. Subgroup difference analysis (Figure 5) demonstrated better therapeutic effects on pain relief for high ACSM adherence. Within the high ACSM adherence subgroup, the individual study heterogeneity for pain was 84%, while in the low or uncertain ACSM adherence subgroup, it was 69.2%. The funnel plot analysis (Figure 9B) indicated approximate symmetry on both sides, suggesting no obvious publication bias. Sensitivity analysis (Figure 10B) revealed that no single study significantly impacted the overall results, further supporting the robustness of our findings.

#### 3.4.3 Mental health

**FIGURE 6:**
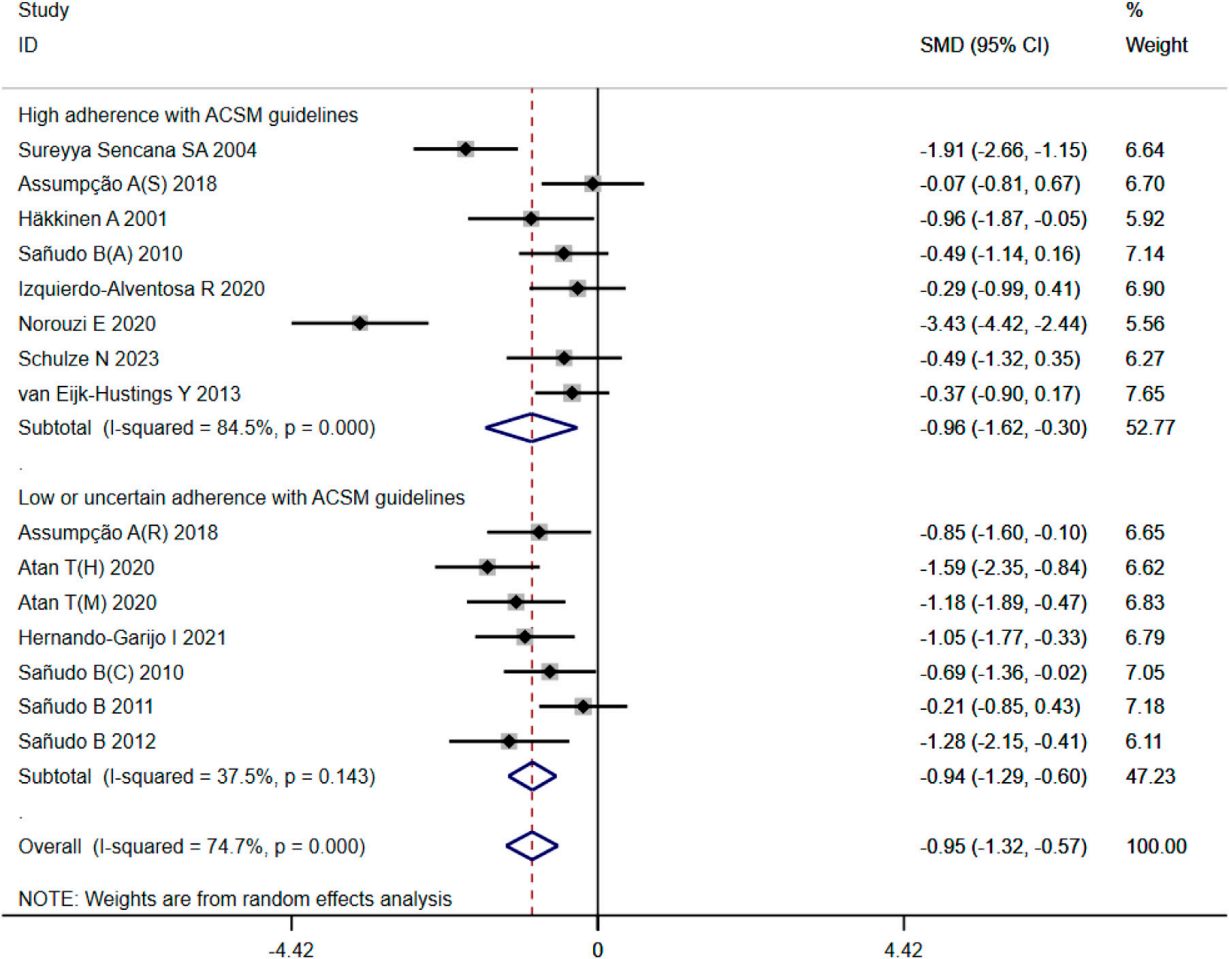
Forest plot of meta-analysis on the effect of exercise intervention and ACSM adherence on mental health in FMS patients.

A total of 525 participants from 15 studies were included in our analysis of the effects of exercise intervention on mental health in FMS patients. A heterogeneity test was conducted, revealing I^2^ to be greater than 50% (I^2^ = 74%, *p* < 0.00001). Consequently, a random effects model was employed for statistical analysis. Our findings indicated a significant beneficial effect of exercise intervention on mental health in FMS patients, with a total combined SMD of −0.95 (95%CI −1.32, −0.57). Subgroup analysis was conducted based on the level of adherence with ACSM recommendations. The combined SMD for the subgroup with high ACSM adherence was −0.96 (95%CI −1.62, −0.30), while for the subgroup with low or uncertain ACSM adherence, the combined SMD was −0.94 (95%CI −1.29, −0.60). Subgroup difference analysis demonstrated no significant difference between exercise interventions with high ACSM adherence and those with low or uncertain ACSM adherence (Figure 6). Therefore, we conclude that exercise interventions with high ACSM adherence do not yield better therapeutic effects on mental health in FMS patients compared to those with low or uncertain ACSM adherence. In the subgroup with high adherence, there was a heterogeneity of 84% among the individual studies measuring mental health. In the low or uncertain adherence subgroup, heterogeneity was 37.5%. Funnel plot inspection (Figure 9C) showed approximate symmetry on both sides, indicating no obvious publication bias. Sensitivity analysis (Figure 10C) indicated that no single study significantly influenced the overall results, thus confirming the robustness of our findings.

#### 3.4.4 Sleep quality

**FIGURE 7:**
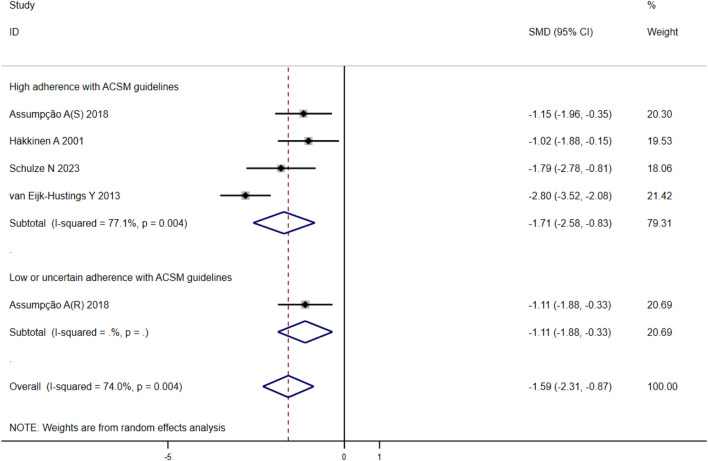
Forest plot of meta-analysis on the effect of exercise intervention and ACSM adherence on sleep quality in FMS patients.

When analyzing the outcome of sleep quality in 169 participants from five studies, we observed significant heterogeneity (I^2^ = 74%, *p* = 0.004) during heterogeneity testing. Therefore, we utilized a random effects model for statistical analysis. Our analysis revealed a total combined SMD of −1.59 (95% CI −2.31, −0.87), indicating a beneficial effect of exercise intervention on the sleep quality of FMS patients. To further explore the data, we conducted subgroup analysis based on the proportion of adherence with ACSM recommendations. In the subgroup with high ACSM adherence, the combined SMD was −1.71 (95% CI −2.58, −0.83), while in the subgroup with low or uncertain ACSM adherence, the SMD was −1.11 (95% CI −1.88, −0.33). Subgroup difference analysis indicated a significant distinction between exercise interventions with high ACSM adherence and those with low or uncertain ACSM adherence (Figure 7). Therefore, we conclude that exercise interventions with high ACSM adherence have superior therapeutic effects on sleep quality scores in FMS patients compared to those with low or uncertain ACSM adherence. Within the subgroup with high ACSM adherence, the heterogeneity was 77%. Examination of the funnel plot (Figure 9D) displayed approximate symmetry on both sides, suggesting no evident publication bias. Sensitivity analysis (Figure 10D) indicated that no single study significantly influenced the overall results, affirming the robustness of our findings.

#### 3.4.5 Fatigue

**FIGURE 8:**
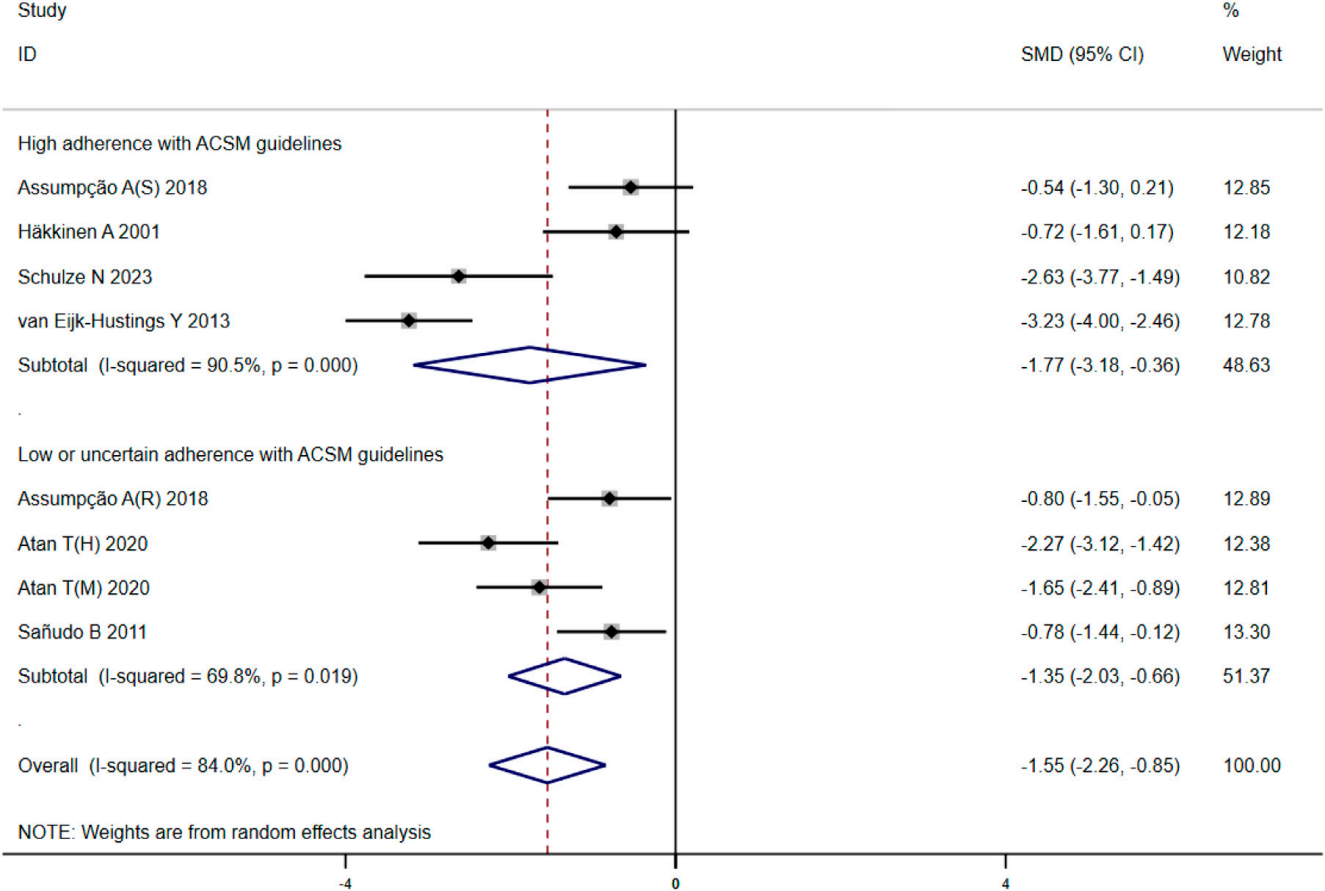
Forest plot of meta-analysis on the effect of exercise intervention and ACSM adherence on fatigue in FMS patients.

**FIGURE 9:**
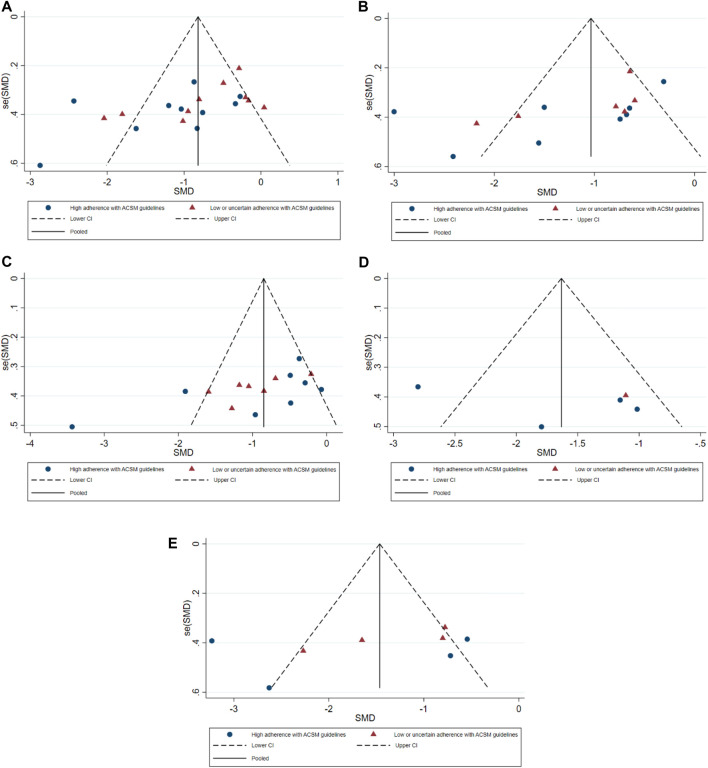
Funnel plot of meta-analysis on the effect of exercise intervention and ACSM adherence on the overall state of health **(A)**, pain **(B)**, mental health **(C)**, sleep quality **(D)**, and fatigue **(E)** in FMS patients.

**FIGURE 10:**
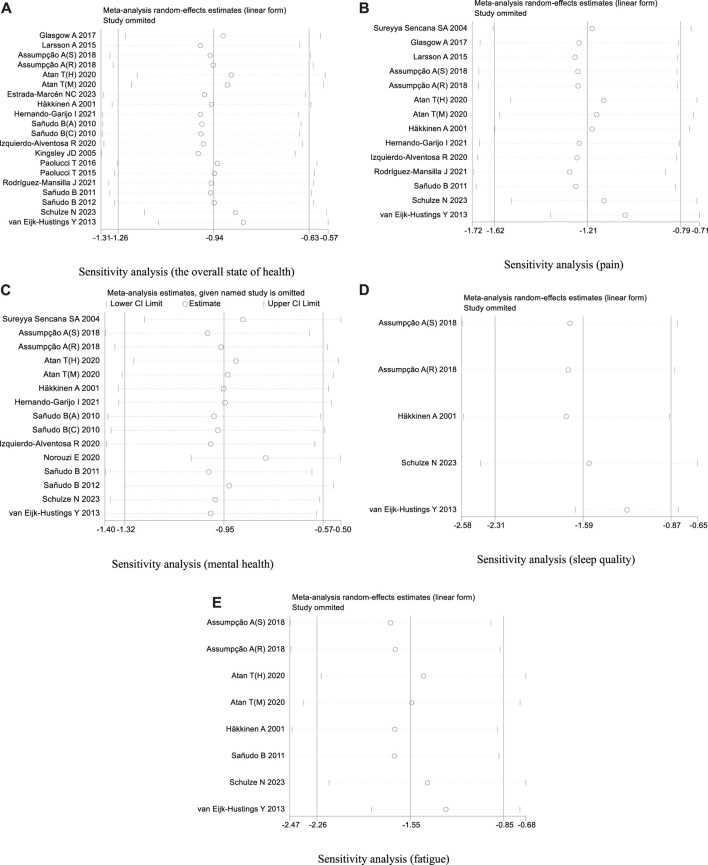
**(A)** Sensitivity analysis (the overall state of health). **(B)** Sensitivity analysis (pain). **(C)** Sensitivity analysis (mental health). **(D)** Sensitivity analysis (sleep quality). **(E)** Sensitivity analysis (fatigue).

After analyzing the outcome of fatigue in 279 participants from eight studies, we observed significant heterogeneity during heterogeneity testing (I^2^ = 84.0%, *p* < 0.001), so we utilized a random effects model for statistical analysis. Our analysis revealed a total combined SMD of −1.55 (95% CI −2.26, −0.85), indicating a beneficial effect of exercise intervention on the fatigue of FMS patients. To further explore the data, we conducted subgroup analysis based on the proportion of adherence with ACSM recommendations. In the subgroup with high ACSM adherence, the combined SMD was −1.77 (95% CI −3.18, −0.36), while in the subgroup with low or uncertain ACSM adherence, the combined SMD was −1.35 (95% CI −2.03, −0.66) (Figure 8). Subgroup difference analysis indicated a distinction between exercise interventions with high ACSM adherence and those with low or uncertain ACSM adherence. In conclusion, the findings suggest that adherence to ACSM recommendations for exercise intervention may help relieve fatigue in patients with FMS. The results of this analysis indicate that adhering to ACSM recommendations for exercise interventions can have a superior therapeutic effect on relieving fatigue compared to low or uncertain adherence to these recommendations. Examination of the funnel plot (Figure 9E) and sensitivity analysis (Figure 10E) support the robustness of these findings.

## 4 Discussion

The ACSM is widely recognized as a leading authority in sports medicine, playing a crucial role in managing various chronic diseases through its evidence-based exercise guidelines (Cui, et al., 2023; Giorgi, et al., 2023; Xijuan, et al., 2023; Yamei, et al., 2023). In 2017, Moseng et al. conducted research demonstrating that patients who adhered closely to the recommended exercise intensities outlined by ACSM experienced more significant pain relief from hip osteoarthritis compared to those with lower adherence (Moseng, et al., 2017). This finding reinforces the importance of maintaining exercise intensity in accordance with ACSM guidelines for effective management of chronic motion diseases.

In this review, comprehensive assessment tools, including the FIQ, were utilized to evaluate multiple health dimensions. These dimensions encompassed the overall state of health and secondary outcomes such as pain intensity, mental wellbeing, sleep quality, and fatigue levels, which exert a significant influence on the overall quality of life experienced by individuals with FMS. Our findings suggest that higher adherence levels to ACSM guidelines resulted in superior pain relief outcomes compared to lower adherence levels. We consider that this improvement in pain relief can be attributed to movement-based interventions which enhance muscle strength and mitigate muscle loss (Estévez-López, et al., 2021). Muscle gain is an effective strategy for pain management in patients with FMS (Gavi, et al., 2014). Furthermore, exercise stimulates the production of endogenous opioids and β-endorphins, which activate descending nociceptive inhibitory mechanisms and result in hypoalgesia (Tan, et al., 2022). Additionally, we observed some research such as Atan T’s study (Atan, et al., 2020) shows a high negative effect, demonstrated higher negative effects, SMD = −2.18 (95%CI −3.01, −1.34) in high-intensity interval training (HIIT) and [SMD = −1.76 (95%CI −2.54, −0.98)] in moderate intensity continuous training (MICT), suggesting that the low-adherence group may also experience improved pain relief in specific cases; however, overall outcomes were not as favorable as those observed in the high-adherence group. Similarly, this phenomenon was reflected in fatigue and mental health outcomes (Figure 6; Figure 8). Their interventions all included aerobic, resistance, and flexibility exercises, but their higher frequency (5 days per week) and intensity (80%–95% of peak HR) resulted in a low ACSM adherence group (Table 5). Sensitivity analysis showed that the results of Atan T were stable (Figure 10B). We considered that a combination of exercise modalities might be more effective in improving the symptoms of FMS in patients with low adherence to ACSM guidelines. This may be due to the combined effect of these exercise modalities, which jointly improve the physical condition of patients through different mechanisms, thus achieving better symptom relief. However, subgroup analyses were not performed because of the limited number of included studies. In terms of fatigue relief, the ACSM high adherence group also showed some advantages. Physiologically, a wide range of central nervous system aberrations have been documented in FMS(Wood, et al., 2009; González-Roldán, et al., 2016), including metabolic and structural abnormalities in the hippocampus, dysfunction of the hypothalamic-pituitary-adrenal axis, and sympathetic hyperactivity (Martinez-Lavin, 2007). These alterations contribute to the elevated levels of fatigue observed in patients with FMS. Exercise has demonstrated potential to reverse these abnormalities by modulating metabolite levels and promoting angiogenesis, neurogenesis, and hippocampal connectivity, thereby potentially alleviating fatigue (Valim, et al., 2013). Furthermore, our meta-analysis reveals that exercise exerts a favorable effect on sleep quality among patients with FMS. This improvement may be attributed to reduced sympathetic tone and increased parasympathetic activity induced by exercise, leading to muscle and nerve relaxation. Consequently, stress is reduced and sleep quality is enhanced (Petzke, et al., 2000; Sarzi-Puttini, et al., 2006). It should be noted that chronic pain is directly associated with poor sleep quality which impacts psychological states such as depression and anxiety (Stults-Kolehmainen, et al., 2014). A cross-sectional study involving 233 patients with chronic pain reported that 36% experienced depression while 66.1% had poor sleep quality (Alhalal, et al., 2021). Therefore, this emphasizes once again the diversity and relevance of physiological mechanisms underlying FMS.

Interestingly, no significant differences were observed between the high and low adherence groups in terms of mental health outcomes. This unexpected finding may suggest that while exercise generally has a positive impact on mental health by reducing symptoms of depression and anxiety, it remains unclear how strict adherence to the ACSM’s exercise recommendations specifically influences these outcomes (Williams, et al., 2011; Krupa, et al., 2024). This complexity could be attributed to the multifactorial nature of mental health problems, which extend beyond exercise adherence (Lima, et al., 2017; Zabihiyeganeh, et al., 2023). For instance, Daniel Rodriguez-Almagro’s study demonstrated that PEBT was most effective in improving SF-36 psychological domain scores when conducted between 21 and 40 sessions [SMD = 0.51 (95%CI 0.28, 0.73)] (Rodríguez-Almagro, et al., 2023). These findings imply that the overall duration of exercise therapy may also influence various outcome measures related to FMS. Similarly, Atan T’s study revealed a favorable effect on mental health after completing five sessions per week for up to 6 weeks (Atan, et al., 2020). However, the Sanudo B’s study implemented twice a week intervention for a duration of up to 6 months (Sañudo, et al., 2012), while the Hernando-Garijo I’s study encompassed 30 sessions (Hernando-Garijo, et al., 2021) and the Izquierdo-Alventosa R’s study involved 16 sessions (Izquierdo-Alventosa, et al., 2020). Studies with different durations of intervention have yielded different results, Sanudo B 2012 for [SMD = −1.28 (95%CI −2.15, −0.41)], Hernando-Garijo I for [SMD = −1.05 (95%CI −1.77, −0.33)], Izquierdo Alventosa R for [SMD −0.29 (95%CI −0.99, 0.41)]. It is plausible that the duration of intervention may have influenced the final results, as shorter sessions (e.g., lasting only for16 sessions) might not fully harness the potential benefits of exercise interventions, leading to smaller effects sizes. How long the improvement in mental health and other measures becomes stable after the intervention is a direction for future research. This may explain the lack of significant differences in mental health outcomes between the high-adherence and low-adherence groups. Currently, a multidisciplinary treatment team utilizing cognitive behavioral therapy (CBT) combined with physical therapy is the most effective way to enhance the mental health of patients with FMS(Monticone, et al., 2015; Ploutarchou, et al., 2023). While exercise may have beneficial effects on anxiety and depression, it may be an added advantage to overall symptom improvement (Bennie, et al., 2020; Juan, et al., 2020). In summary, these results demonstrate further diversity and relevance of FMS in physiological mechanisms and provide clinical evidence for understanding potential mechanisms of exercise in human physiological function regulation and chronic disease management. Long-term treatment courses combining multiple modalities are suggested for improving mental health and other symptoms in FMS patients.

Previous randomized controlled trials have demonstrated several advantages of exercise therapy for FMS patients (Zis, et al., 2017; Marcuzzi, et al., 2022). These include improved cardiovascular fitness, increased muscle strength, enhanced flexibility, and better overall physical functioning (Juan, 2019; Ye et al., 2021). Aerobic exercises contribute significantly to cardiovascular health, resistance exercises enhance muscle strength, and flexibility exercises improve range of motion (Haack, et al., 2020; El-Sawy, et al., 2024). Together, these improvements help in managing FMS more effectively, aligning well with the ACSM recommendations (Siracusa, et al., 2021). However, exercise therapy is not without its challenges. For instance, potential confounders such as participants’ baseline health status, mental health, or lifestyle choices could still have an impact on the results. These factors may influence both adherences to exercise regimens and the measured outcomes. For instance, participants with better baseline health or more positive psychological states might find it easier to adhere to exercise guidelines and experience greater benefits. Conversely, those with poorer baseline health or higher levels of psychological distress might struggle more with adherence, thereby affecting their outcomes. Patients with severe limitations or comorbidities may find it difficult to adhere to rigorous exercise regimens (Yiru, et al., 2019; Liu et al., 2020). Additionally, the intensity and frequency required to achieve significant benefits might not be feasible for all types of diseases and patients, raising concerns about safety and practicality. For instance, overexertion can potentially lead to flare-ups in pain and fatigue, counteracting the benefits of exercise (Busch, et al., 2008). Our study categorized ACSM adherence into high, uncertainty, and low levels based on participants’ adherence to prescribed intensity and frequency. High adherence was associated with better physical health outcomes, demonstrating the importance of following structured exercise guidelines. This classification helps in evaluating the effectiveness of exercise dosages tailored to individual capabilities and limitations. To provide a more integrated view of exercise therapy in FMS management, it is essential to consider the therapeutic benefits of different exercise modalities in relation to ACSM guidelines. We suggest adherence to these guidelines ensures that the diverse effects of aerobic, resistance, and flexibility exercises are maximized, leading to comprehensive improvements in health, pain relief, sleep, and fatigue management.

## 5 Limitations

Several limitations were encountered in this study. Firstly, the inclusion of reference papers may introduce selective bias, potentially affecting the generalizability of the study results. Moreover, certain articles retrieved during literature search provided unclear or incorrect definitions of HIIT. Additionally, some studies reported relevant data that were not recorded (NR), which may have introduced errors in our assessment and may have amplified errors in subsequent analyses. To address these issues, we strictly adhered to inclusion and exclusion criteria that specified particular forms of exercise, exercise frequency, and exercise duration. The aggregation of results was performed using a random-effects model to account for interstudy variation. Furthermore, a sensitivity analysis was conducted to identify significant biases in the studies and assess their impact on the overall findings. It is important to note that the choice of adherence threshold can influence the results. To balance methodological rigor with an adequate sample size, we selected a 75% adherence threshold. Setting a threshold of 100% would limit the number of included studies and reduce statistical power and generalizability; however, it could provide valuable insights if achieved under perfect adherence conditions. Conversely, setting a threshold of 50% may include less rigorous studies and dilute observed effects. By choosing a 75% adherence threshold, we included a larger pool of studies that enhanced statistical robustness and methodologic rigor in our findings. Despite the efforts made to address the aforementioned limitations, readers should interpret our results cautiously given the limited availability of research on intervention measures for FMS. Future studies are warranted to further investigate the specific efficacy of different types and intensities of exercise for FMS, as well as determine optimal exercise protocols.

## 6 Conclusion

This meta-analysis demonstrates that adherence to the exercise guidelines of the ACSM significantly enhances the overall state of health, pain, sleep quality, and fatigue management in patients diagnosed with FMS. Importantly, a high level of adherence to ACSM guidelines leads to superior improvements in these domains compared to low or uncertain adherence. These findings suggest that structured exercise programs based on ACSM recommendations can effectively optimize multiple dimensions of health in FMS patients. Furthermore, while exercise positively impacts mental health, our analysis indicates that strict adherence to exercise guidelines does not significantly influence mental health outcomes compared to low adherence, thereby highlighting the intricate role of psychological factors in FMS. The future treatment of patients with FMS should consider incorporating movement-based interventions in combination with other therapeutic modalities to optimize the management of FMS and enhance mental health in patients.

## Data Availability

The original contributions presented in the study are included in the article, further inquiries can be directed to the corresponding author.
